# Docosahexaenoic acid-rich algae oil supplementation on breast milk fatty acid profile of mothers who delivered prematurely: a randomized clinical trial

**DOI:** 10.1038/s41598-021-01017-8

**Published:** 2021-11-02

**Authors:** Hélène Fougère, Jean-François Bilodeau, Pascal M. Lavoie, Ibrahim Mohamed, Iwona Rudkowska, Etienne Pronovost, David Simonyan, Line Berthiaume, Mireille Guillot, Bruno Piedboeuf, Pierre Julien, Isabelle Marc

**Affiliations:** 1 Department of Pediatrics, CHU de Québec-Université Laval, 2705 Boulevard Laurier, Quebec City, QC G1V 4G2 Canada; 2 Department of Medicine, Faculty of Medicine, Université Laval, Quebec City, QC Canada; 3 Endocrinology and Nephrology Research Axis, CHU de Québec-Université Laval, Quebec City, QC Canada; 4 Department of Pediatrics, University of British Columbia, Vancouver, BC Canada; 5 Departments of Pediatrics and Nutrition, CHU Sainte-Justine, University of Montreal, Montreal, QC Canada; 6 Department of Kinesiology, Université Laval, Quebec City, QC Canada; 7 Clinical and Evaluative Research Platform, CHU de Québec-Université Laval, Quebec City, QC Canada

**Keywords:** Paediatric research, Clinical trial design, Lipids

## Abstract

Preterm infants are deficient in long-chain polyunsaturated fatty acids, especially docosahexaenoic acid (DHA), a fatty acid (FA) associated with an increase in bronchopulmonary dysplasia (BPD). In two previous randomized control trials, DHA supplementation did not reduce the risk of BPD. We examined the breast milk FA profile, collected 14 days after birth, of mothers who delivered before 29 weeks of gestation and who were supplemented with DHA-rich algae oil or a placebo within 72 h after birth as part of the MOBYDIck trial. Milk FA were analyzed by gas chromatography. The total amount of FA (mg/mL) was similar in both groups but the supplementation increased DHA (expressed as % of total FA, mean ± SD, treatment vs placebo, 0.95 ± 0.44% vs 0.34 ± 0.20%; P < 0.0001), n-6 docosapentaenoic acid (DPA) (0.275 ± 0.14% vs 0.04 ± 0.04%; P < 0.0001) and eicosapentaenoic acid (0.08 ± 0.08% vs 0.07 ± 0.07%; P < 0.0001) while decreasing n-3 DPA (0.16 ± 0.05% vs 0.17 ± 0.06%; P < 0.05). Supplementation changed the ratio of DHA to arachidonic acid (1.76 ± 1.55% vs 0.60 ± 0.31%; P < 0.0001) and n-6 to n-3 FA (0.21 ± 0.06% vs 0.17 ± 0.04%; P < 0.0001). DHA-rich algae supplementation successfully increased the DHA content of breast milk but also included secondary changes that are closely involved with inflammation and may contribute to changing clinical outcomes.

## Introduction

Preterm infants have limited anti-inflammatory defenses during the early neonatal period^1^, which contributes to the development of morbid diseases including bronchopulmonary dysplasia (BPD)^2,3^. This chronic lung disease affects approximately half of very preterm infants^4^. BPD is characterized by reduced postnatal pulmonary development found to be associated with an abnormal inflammatory response^5^. Optimal nutrition is fundamental to support the needs of preterm infants and prevent BPD. Breast milk is rich in long-chain polyunsaturated fatty acids (i.e. which have a chain with more than 18 carbons; LC-PUFA), which are precursors of bioactive molecules involved in inflammation processes^6^. The two essential LC-PUFA linoleic acid (C18:2 n-6; LA) and alpha-linolenic acid (C18:3 n-3; ALA) can be further elongated and desaturated in the endoplasmic reticulum of cells, respectively into arachidonic acid (C20:4 n-6; AA) and eicosapentaenoic acid (C20:5 n-3; EPA) then docosahexaenoic acid (C22:6 n-3; DHA). The LC-PUFA derived metabolites have different roles in the inflammation process. The DHA, AA and EPA influence the inflammatory response, in part through the formation of specialized pro-resolving mediators that play an essential role in initiating the inflammation resolution cascade^7^. Pro-resolving mediators, generated from these LC-PUFA may act simultaneously but distinctively in attenuating inflammation during BPD^3^. For example, DHA may have direct anti-inflammatory properties, while AA is a substrate for the production of pro-inflammatory eicosanoids, hence high AA has been linked to increased inflammation^7^. Therefore, inflammation homeostasis depends in part on the balance between n-6 and n-3 fatty acids (FA). Furthermore, DHA plays a predominant role in neurogenesis and brain development during pregnancy and first years of life because it is crucial for the metabolism, growth, and differentiation of neurons^8^. In very preterm infants, the low in utero DHA accretion to the brain is hypothesized to contribute to the increased prevalence of neurodevelopmental deficits. The review of Gould et al.^9^ on the influence of DHA supplementation (in pregnancy, the neonatal period, and infancy) on child behavioral functioning showed that a few studies reported a potentially adverse effect of DHA on behavior. However, the evidence is insufficient to reach a definitive conclusion regarding the effect of early DHA interventions on child behavior.

Preterm infants have a limited capacity for elongating and desaturating the essential fatty acids to LC-PUFA, and negligible LC-PUFA stores in adipose tissue, especially DHA and AA^10^. Low levels of DHA and AA have been associated with adverse neonatal outcomes, particularly in very preterm infants^11,12^. In an experimental animal model, supplementation with n‐3 LC-PUFA through an enriched diet administered to the mother during lactation, was found beneficial to lung architecture^13^. Human observational studies have shown that low levels of DHA correlate with an increased risk of chronic lung disease in preterm infants^11^. A supply of LC-PUFA through diet is therefore necessary and breast milk is an effective vehicle for increasing DHA stores in plasma and tissues^14^. However, the best nutritional way to achieve this balance in preterm infants remains unclear.

The DINO trial suggested a reduction in BPD, as defined by supplemental oxygen needs, in preterm infants following a maternal DHA supplementation^15^. On the other hand, two recent randomized clinical trials reported that supplementing DHA to preterm infants either directly through fish oil supplementation^16^, or via maternal algae oil supplementation^17^, increased BPD. To help reconcile these findings, we *hypothesize* that enteral DHA either administered directly to the preterm infant or through breast milk from their mothers adversely affects the inflammatory response and consequently contributes to BPD development.

To shed some light on this question, we presume that supplementing mothers of very preterm infants (delivery before 29 weeks of gestation) with DHA-rich algae oil during the first weeks of lactation affects various breast milk FA, in particular LC-PUFA precursors of molecules with biological activities, and not specifically the DHA.

This study is a secondary analysis of data from the MOBYDIck trial^17^. The objective was to characterize the breast milk FA profile among mothers who delivered very prematurely (before 29 weeks of gestation) after a neonatal DHA-rich algae oil supplementation compared to a control group. The signature of FA profile following supplementation with DHA-rich algae oil could provides insights on the unexpected deleterious effect of this supplementation on BPD observed previously.

## Results

### Maternal characteristics and compliance to the intervention

**Figure 1:**
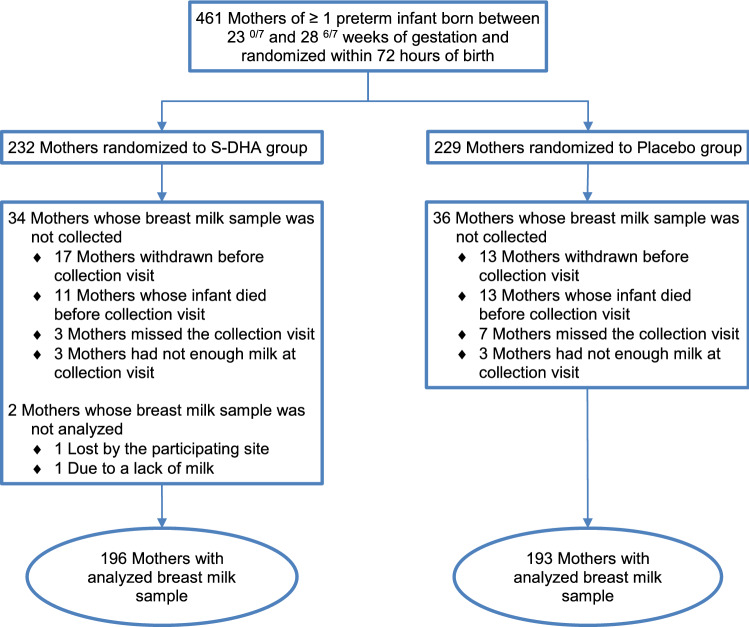
Trial flow diagram of mothers in the group supplemented in DHA (S-DHA) and the placebo group.

**Figure 2:**
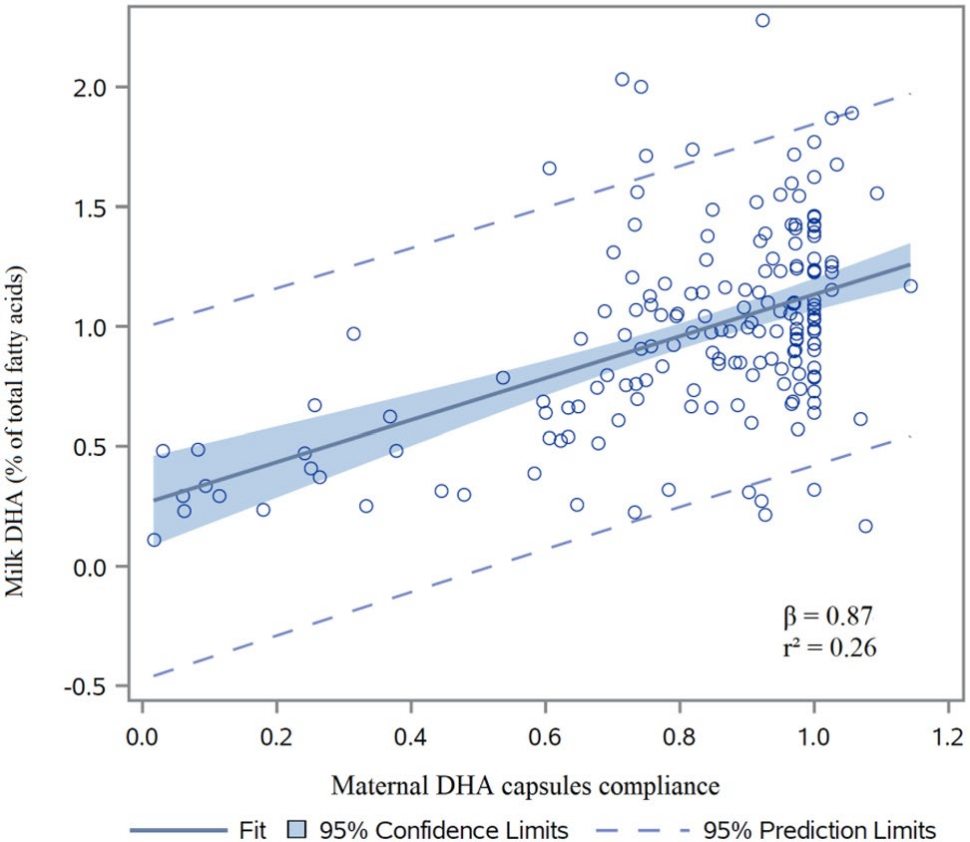
Milk DHA content in function of the maternal compliance in mothers who received DHA capsules. Compliance corresponds to the ratio of capsules taken over expected from randomization until day 14 ± 2 after delivery. *DHA* docosahexaenoic acid.

From 461 mothers enrolled in the MOBYDIck trial, breast milk samples were analyzed for 389 mothers (84.4%), with 196 mothers in the S-DHA group and 193 mothers in the placebo group (Fig. 1). Characteristics of mothers who provided breast milk samples did not differ between the two groups (Table 1). Results for compliance (ratio of capsules taken over expected from randomization until the time of breast milk samples) as measured by counting capsules returned by mothers at the time of breast milk sample collection (day 14) were respectively 82 ± 24% in the S-DHA group and 78 ± 27% in the placebo group. There was a linear influence of maternal DHA capsule compliance on breast milk DHA levels of mothers from the S-DHA group (β = 0.87, r^2^ = 0.26; P < 0.0001; Fig. 2). There was also a linear effect of maternal dietary DHA intake on breast milk DHA levels of mothers in the placebo group (β = 0.00056, r^2^ = 0.18; P < 0.0001), but not in the S-DHA group (β = 0.000318, r^2^ = 0.08; P = 0.11).

**Table 1:** Characteristics of mothers whose breast milk samples were analyzed in the group supplemented in DHA (S-DHA) and in the placebo group.

Characteristic	Mean ± SD^a^		*P*-value^b^
	S-DHA (n = 196)	Placebo (n = 193)	
**Pre-pregnancy**			
Weight, kg^c^	70.9 ± 16.1	73.0 ± 19.8	0.59
Body mass index, kg/m^2c^	26.5 ± 5.9	27.4 ± 7.3	0.66
**At delivery**			
Age, year	30.9 ± 5.3	31.1 ± 5.3	0.71
Gestational age, week	26.7 ± 1.4	26.5 ± 1.6	0.15
Parity^c^	1.70 ± 0.91	1.85 ± 1.13	0.22
Maternal dietary DHA intake, mg/day^c,d^	191 ± 261	189 ± 237	0.60
**After delivery**			
Ratio of capsules taken over expected from randomization until day 14 ± 2 after delivery^c^	0.82 ± 0.24	0.78 ± 0.27	0.11

*DHA* docosahexaenoic acid.

^a^Unless otherwise indicated.

^b^P-values were estimated by the Mann–Whitney–Wilcoxon test.

^c^There was missing data (< 10%).

^d^Mean in the last month before randomization.

### Breast milk FA composition

The treatment did not change the total amount of FA in milk in the S-DHA group compared with the placebo group (35.3 ± 12.0 mg/mL vs 36.7 ± 13.4 mg/mL; P = 0.31).

Breast milk FA contents, expressed as percentages by weight of total detected FA, are reported in Table 2. DHA content was higher in the S-DHA group compared to the placebo group (0.95 ± 0.44% vs 0.34 ± 0.20%; P < 0.0001).

**Table 2:** Fatty acid composition of maternal breast milk measured at 14 days after delivery in the group supplemented in DHA (S-DHA) and in the placebo group.

Fatty acid composition expressed as % of total fatty acids	Mean ± SD		*P*-value^a^
	S-DHA (n = 196)	Placebo (n = 193)	
*Cis-*9 16:1^b^	1.87 ± 0.64	2.01 ± 0.65	*
*Cis-*11 18:1^b^	1.89 ± 0.40	1.99 ± 0.40	**
*Trans-*9 18:1^b^	0.14 ± 0.11	0.12 ± 0.09	0.26
*Trans-*11 18:1^b^	0.25 ± 0.18	0.21 ± 0.14	*
**n-6 fatty acids**			
18:2 n-6 (LA)	12.6 ± 2.72	13.0 ± 2.70	0.15
18:3 n-6	0.08 ± 0.06	0.08 ± 0.06	0.90
20:2 n-6	0.43 ± 0.10	0.45 ± 0.09	*
20:3 n-6	0.51 ± 0.16	0.52 ± 0.17	0.37
20:4 n-6 (AA)	0.57 ± 0.14	0.57 ± 0.16	0.96
22:2 n-6	0.06 ± 0.03	0.06 ± 0.03	0.13
22:4 n-6	0.14 ± 0.05	0.15 ± 0.06	0.20
22:5 n-6 (n-6 DPA)	0.27 ± 0.14	0.04 ± 0.04	***
**n-3 fatty acids**			
18:3 n-3 (ALA)	1.51 ± 0.50	1.53 ± 0.52	0.77
18:4 n-3	0.10 ± 0.05	0.10 ± 0.05	0.99
20:3 n-3	0.09 ± 0.06	0.09 ± 0.04	0.76
20:4 n-3	0.15 ± 0.07	0.14 ± 0.08	0.31
20:5 n-3 (EPA)	0.08 ± 0.05	0.07 ± 0.07	***
22:5 n-3 (n-3 DPA)	0.16 ± 0.05	0.17 ± 0.06	*
22:6 n-3 (DHA)	0.95 ± 0.44	0.34 ± 0.20	***
**Ratio**			
DHA:n-6 DPA	3.80 ± 1.06	7.14 ± 3.56	***
DHA:AA	1.76 ± 1.55	0.60 ± 0.31	***
n-3:n-6	0.21 ± 0.06	0.17 ± 0.04	***
*Trans*-11 18:1 / *cis*-11 18:1	0.14 ± 0.11	0.11 ± 0.08	**
**Sum**			
∑Saturated	44.8 ± 5.74	44.4 ± 6.21	0.43
∑MUFA	37.4 ± 4.51	38.2 ± 4.97	*
∑PUFA n-6	14.6 ± 2.84	14.8 ± 2.80	0.42
∑PUFA n-3	3.04 ± 0.76	2.45 ± 0.71	***
∑*Cis* fatty acids	54.4 ± 5.84	54.9 ± 6.24	0.40
∑*Trans* fatty acids	0.47 ± 0.29	0.42 ± 0.21	0.14

*LA* linoleic acid, *ALA* alpha-linolenic acid, *AA* arachidonic acid, *DPA* docosapentaenoic acid, *EPA* eicosapentaenoic acid, *DHA* docosahexaenoic acid, *MUFA* monounsaturated fatty acid, *PUFA* polyunsaturated fatty acid.

^a^Estimated by the Mann–Whitney–Wilcoxon test. *P < 0.05; **P < 0.01; ***P < 0.0001.

^b^For these FA, a nomenclature of the *cis*/*trans* type instead of the n nomenclature was chosen to discuss the results.

In addition to increasing DHA content, the algae oil supplementation increased n-6 docosapentaenoic acid (C22:5 n-6; DPA) content in the S-DHA group compared to the placebo group (0.27 ± 0.14% vs 0.04 ± 0.04%; P < 0.0001). These variations of DHA and n-6 DPA resulted in a decrease of the DHA-to-n-6 DPA ratio in the S-DHA group compared to the placebo group (3.80 ± 1.06% vs 7.14 ± 3.56%; P < 0.0001; Table 2). Among n-3 LC-PUFA, content of both EPA and n-3 DPA (C22:5 n-3) were modified with the supplementation: EPA was increased in the S-DHA group compared to the placebo group (0.08 ± 0.05% vs 0.07 ± 0.07%; P < 0.0001), while n-3 DPA was decreased (0.16 ± 0.05% vs 0.17 ± 0.06%; P < 0.05). Among n-6 LC-PUFA, AA content was similar between the two groups, but the ratio of DHA-to-AA was significantly higher in the S-DHA group compared to the placebo group (1.76 ± 1.55% vs 0.60 ± 0.31%; P < 0.0001). The total amount of n-3 LC-PUFA was significantly different between the S-DHA group and the placebo group (3.04 ± 0.76% vs 2.45 ± 0.71%; P < 0.0001), while the combined amount of n-6 LC-PUFA was similar in both groups (14.6 ± 2.84% vs 14.8 ± 2.80%; P = 0.42). These variations in the content of LC-PUFA, mainly n-3, led to a different ratio of n-3-to-n-6 between the S-DHA and the placebo groups (0.21 ± 0.06% vs 0.17 ± 0.04%; P < 0.0001).

Content of cis-9 16:1 was decreased in the S-DHA group compared to the placebo group (1.87 ± 0.64% vs 2.01 ± 0.65%; P < 0.05), as well as the content of cis-11 18:1 (1.89 ± 0.40% vs 1.99 ± 0.40%; P < 0.01), while trans-11 18:1 was increased in the S-DHA group compared to the placebo group (0.25 ± 0.18% vs 0.21 ± 0.14%; P < 0.05).

## Discussion

In the present study, we reported a complete breast milk FA profile following a DHA rich-algae oil supplementation as a secondary analysis of data from the MOBYDIck trial, conducted in a large number of mothers of very preterm infants. As expected, the supplementation of mothers of very preterm infants with 1200 mg/day of DHA during lactation, in the neonatal period, significantly increased DHA content in breast milk at day 14 post-delivery compared to mothers receiving a placebo. However, the DHA-rich algae oil supplementation also significantly altered other FA in breast milk, especially n-6 DPA and the n-3 FA EPA and DPA, leading to an increase of n-3-to-n-6 and DHA-to-AA ratios.

Although maternal diet seems to have little impact on the milk fat content, it is well known that the diet influences the milk FA composition regardless of their origin (FA synthesized by the mammary gland or taken from maternal plasma)^18^. Furthermore, contrary to n-6 FA, the intake of n-3 FA is low in women during pregnancy and lactation, which is typical in a western country’s diets^19^. These particularly low maternal intake of n-3 PUFA resulted in a significant reduction of DHA content in newborn erythrocytes and breast milk, and thus reduced the DHA availability for the infant^20^. As mentioned in the results, there was a linear effect of maternal dietary DHA intake on breast milk DHA levels of mothers in the placebo group. However, this correlation was not found in the S-DHA group, but breast milk DHA levels of mothers were correlated with the maternal DHA supplementation compliance, suggesting that the effects of DHA supplementation taken by the mother subsided the effect of DHA of the diet.

The effect of a DHA supplementation at a specified dose (alone or in association with other LC-PUFA) on the breast milk FA composition has been reported by a few small randomized studies, which allows us to consider our results in the context of previous studies (Supplementary Table S1). Among them, 2 studies specifically investigated milk from mothers of preterm infants^11,21^, but included fewer participants than MOBYDIck (10 and 98 respectively). These two studies were also conducted in populations of western countries. Among the 17 identified studies, only one used LC-PUFA from algae^11^, while all others used LC-PUFA from fish (krill, salmon, tuna, cod) mainly in the form of oil. In most cases, these supplements were used to increase the intake of DHA and/or EPA. The doses of DHA provided in these studies were close to the daily recommendations (200–250 mg/day); however, 5 studies reported the effects on the milk FA profile of a dose similar to the current study, i.e. greater than 800 mg/day.

Half of the studies began treatment at the same time as the present study: after delivery (at week 2 of lactation at the earliest). For the others, treatment started before delivery (at week 14 of gestation at the earliest). Only one study started treatment at the day of birth^11^. Duration of treatment varied greatly between the studies, ranging from 1 week to several months. Depending on the studies, milk samples used for FA profile analysis were collected from day 1 to several months after birth, but in most cases (n = 11), milk samples were collected during the first weeks of lactation (weeks 1 to 4). These variations between studies could partly explain the differences in milk FA profile observed. Indeed, the DHA content in the milk samples of the placebo group is very variable from one study to another. For the supplemented group, although there was some variability in the response, in general, a higher dose of DHA in the supplementation led to a higher DHA content in milk. Indeed, milk DHA contents of 1 to 1.4% were reported following a DHA supplementation between 900 and 1021 mg/day (Supplementary Table S1). However, a significant number of factors, such as maternal characteristics, including ethnicity^22^, or differences in experimental design, can explain these variations. In the current study, the milk DHA content (0.95%) obtained in response to DHA-algae oil supplementation is slightly lower than what has been reported in the literature^21,23,24^, but this value is affected by the mother’s compliance to the treatment, which is often not reported in these studies containing small numbers of participants. Finally, a supplementation of 1200 mg/day of DHA allows for breast milk DHA contents to be considerably higher than those obtained with the recommendations (200–250 mg/day), increasing the possibility of improving the DHA intake significantly for preterm infants.

In addition to DHA, the effect of such supplementation on milk EPA content, must be considered because of its important role in many metabolic and physiological processes in human nutrition and health^25^. Most studies using supplementation providing DHA and EPA reported significant increases in milk EPA content compared to placebo (Supplementary Table S1). In the current study, even if algae oil capsules contained low EPA content (1.15%; Table 3), a significant increase in breast milk EPA was observed compared with the placebo group (+ 15%). This could be due to the presence of ALA in the supplemented group, which is a precursor of n-3 LC-PUFA, promoting the biosynthesis of EPA in addition to EPA provided by the capsules. Indeed, despite the presence of a retroconversion pathway of DHA into EPA in humans, increases in plasma and tissue EPA levels following DHA supplementation are not the result of increased retroconversion^26^. In literature, studies reported variable effects on breast milk EPA content, but as for DHA, milk EPA content increase with the dose used in the supplementation. For example, supplementation in tuna oil providing an EPA dose of 195 mg/day, resulted in a milk EPA content of 0.1%^21^, while a fish oil supplementation of 1300 mg/day of EPA, resulted in a milk EPA content of 0.7%^23^.

**Table 3:** Fatty acid composition of the placebo and DHA-rich algae oil capsules.

Fatty acid composition expressed as % the total fatty acids	DHA-rich algae oil capsule	Placebo capsule
14:0	5.90	0.07
16:0	17.0	10.7
*Cis-*9 16:1	0.10	0.10
18:0	0.65	3.38
*Trans*-9 18:1	0.18	0.00
*Cis*-9 18:1	6.59	25.9
*Cis*-11 18:1	0.20	1.34
*Cis*-9,*tran*s-12 18:2	0.0	0.26
*Trans*-9,*cis*-12 18:2	0.0	0.21
20:0	0.12	0.40
20:1 n-9	0.00	0.22
22:0	0.14	0.36
24:0	0.24	0.15
**n-6 fatty acids**		
18:2 n-6 (LA)	0.80	52.4
18:3 n-6 (ALA)	0.31	0.03
20:3 n-6	0.46	0.0
20:4 n-6 (AA)	0.48	0.0
22:4 n-6	0.13	0.0
22:5 n-6 (n-6 DPA)	19.0	0.02
**n-3 fatty acids**		
18:3 n-3	0.0	4.49
18:4 n-3	0.36	0.01
20:4 n-3	0.96	0.0
20:5 n-3 (EPA)	1.15	0.0
22:3 n-3	0.01	0.0
22:5 n-3 (n-3 DPA)	0.59	0.0
22:6 n-3 (DHA)	44.6	0.06
**Ratio or sum**		
∑n-3/∑n-6	2.25	0.09
DHA:n-6 DPA	2.35	3.0
∑n-6	21.2	52.9
∑n-3	47.7	4.56
∑Saturated	24.0	15.1
∑*Trans* fatty acids	0.18	0.47
∑*Cis* fatty acids	75.8	84.5

*LA* linoleic acid, *ALA* alpha-linolenic acid, *AA* arachidonic acid, *DPA* docosapentaenoic acid, *EPA* eicosapentaenoic acid, *DHA* docosahexaenoic acid.

In the present study, the DHA-rich algae oil supplementation led to an increase in milk n-6 DPA compared with the placebo group, which is probably due to the n-6 DPA contained in the algae-oil capsules representing 19% of total FA (versus 0.02% in placebo capsules; Table 3), but a fraction from endogenous synthesis cannot be excluded. The study of Valentine, et al.^11^ reported that the effects of algae oil supplementation on breast milk FA did not result in an increase in n-6 DPA (Supplementary Table S1). Interestingly, the study of Jensen et al.^27^ where a maternal supplementation in fish oil was used to bring 260 mg/day of DHA also reported a significant increase in n-6 DPA compared with a placebo (+ 50%). This could indicate an inadequate n-3 LC-PUFA status, as the supplement did not contain n-6 DPA; generally, this LC-PUFA tends to increase if adequate amounts of DHA are not available from either dietary intake or biosynthesis^27^.

Otherwise, in the current study, supplementation in algae oil led to a decrease in n-3 DPA, which contradicts data reporting an increase in n-3 DPA following supplementation with a similar dose of DHA^21,23,24,28^. However, it should not be overlooked that in these studies, this FA came from the supplement itself. Of note, all these studies used fish supplements rather than algae.

Although reported contents are very low, we observed an unexpected increase in trans-11 18:1 in breast milk from mothers in the S-DHA group. It has been shown that trans-FA could have negative effects on human health^29^. However, trans-FA have 2 origins; they can either be formed from natural cis-unsaturated FA during a process called biohydrogenation in the rumen (trans-FA from ruminants), or through the partial hydrogenation of vegetable oils (industrial trans-FA)^30^. The latter trans-FA are particularly associated with cardiovascular disease risk factors^31^. In the current study, only ruminants’ trans-11 18:1 was found to be increased in the S-DHA group, which is less concerning. Trans-FA are known to decrease the biosynthesis of LC-PUFA, such as AA and DHA, by inhibiting the activity of the ∆-6 desaturase enzyme, from essential FA^32^. Moreover, Koletzko^33^, reported an inverse correlation between total trans-FA and the contents of LC-PUFA in plasma lipid fractions in preterm infants 4 days after birth, and trans-FA were also inversely correlated with birth weight. We suspect that the increase in the S-DHA group is most likely due to a different content of trans-FA in diet between supplemented and placebo groups. Even though groups were randomized and appeared well balanced in terms of diet, we did not have precise data regarding the intake of dairy products, which could have confirmed this hypothesis. Furthermore, none of the studies reporting the effects of DHA supplementation (alone or combined with other LC-PUFA) mention any variations in these trans-FA in milk (Supplementary Table S1). A study has shown that the rate of trans-FA in mature milk was 1.5 ± 0.2%^34^, approximately 3 times more than in the current study. Therefore, the effect of trans-FA on the biosynthesis of LC-PUFA appears limited in the current study.

The main FA ratio modified by the DHA supplementation was the ratio n-3-to-n-6, which reached a recommended value. It is believed that this ratio must be at least 1:5 in order to not influence the stimulation or suppression of physiologically important processes in the body^35^. Otherwise, in the current study, the ratio of DHA to AA was strongly modified by the supplementation, due to a high intake of DHA that was not counterbalanced by an intake of AA. In the literature, it is known that high intakes of DHA result in a decrease of tissue AA^36^. Indeed, previous data has shown that the supplementation in LC-PUFA could be accompanied by a decrease in breast milk AA, by an average of 25%^37^. However, this was not observed in the current study as AA levels remained similar between to two groups after 14 days of supplementation. Nevertheless, when consumed with DHA, n-6 DPA may help to maintain AA levels. Indeed, a study which determined the liver FA composition of rat supplemented in FA, after an essential FA deficiency period, showed that n-6 DPA can be converted to AA^38^. Thus, it is possible that the simultaneous administration of n-6 DPA and DHA in the algae oil supplement was able to cause a slower decline in AA than that normally observed when DHA is administered alone. Contrary to DHA, AA can be a precursor of pro-inflammatory mediators^7^. However, one study in adults showed that an increased intake in AA and LA did not significantly affect inflammatory markers, despite the fact that a high intake of n-6 FA inhibits the anti-inflammatory effect of n-3 FA^39^. The competition between n-6 and n-3 FA in the context of inflammation is very complex and not yet resolved, but several studies have shown the necessity to supplement both DHA and AA rather than DHA alone. Indeed, supplementation of both DHA and AA is necessary to maintain the natural composition of breast milk and to avoid suppressing AA-mediated metabolism^40^. Preterm infants have a very limited ability to convert LA and ALA precursors into AA and DHA respectively. Consequently, their AA and DHA levels are usually low compared to term infants, and they require a higher intake of AA and DHA^41^. In preterm infants, supplementation in DHA and AA can significantly increase the LC-PUFA level. These 2 LC-PUFA are especially essential for both optimal visual and brain development^42^. In a mouse model, Harauma et al.^43^ showed that supplementing AA or DHA alone was insufficient for optimal development. Moreover, insufficient AA intake could reduce the clinical benefits of LC-PUFA on cognitive outcomes^44^.

Thus, based on our findings, the unbalanced high ratio of DHA relative to AA might be a potential explanation for the lack of clinical benefit observed in the MOBYDIck trial on neonatal outcomes including BPD^17^. This does not exclude a direct benefit of supplementing n-3 LC-PUFA alone on neurodevelopmental outcomes, but the reduced availability of AA could have prevented the beneficial effect of DHA. The balance between the DHA and AA on inflammation in BPD and its consequences remains to be defined more precisely.

## Limitations of the study

Data on the mother’s diet were not collected, although diet modulates the milk FA composition. However, an estimation of the maternal DHA intake from marine sources was collected and was similar in both groups. Moreover, the genetic profile was not determined in the trial although genetic variations may have an impact on the milk FA composition. For example, breast milk fatty acids have been shown to be influenced by the genotype of the FADS1 and FADS2 gene cluster. These genotypes have been previously shown to influence ARA, EPA and DHA levels^45^.

## Conclusion

This study is the first to report a complete milk FA profile, in response to DHA supplementation in mothers of preterm infants. The trial was conducted on many participants and showed that supplementing mothers of preterm infants with DHA-rich algae oil is an effective way to increase breast milk DHA content in the context of a western diet (expected to be too low in n-3 LC-PUFA), to address the dietary needs of preterm infants (< 29 weeks of gestation). An intake of 1200 mg/day made it possible to reach a breast milk DHA content of nearly 1% of total FA, which is approximately 3 times more than what is observed without supplementation. The algae-oil supplementation not only increased the DHA, but also increased the n-6 DPA content. Results further indicate that the increased maternal intake of these two FA resulted in the modification of other milk FA content. Consequently, changes in FA ratios, including the DHA to AA ratio, could modify the regulation of inflammation. Further studies are required to better understand how modifications of these LC-PUFA contents in breast milk, as a consequence of the DHA-rich algae supplementation, impact neonatal outcomes. Moreover, in a next step of this work, it will be interesting to determine whether the maternal characteristics may have influenced the FA profile in breast milk.

## Methods

The protocol was approved by Health Canada and the research ethics boards of the CHU de Québec-Université Laval (trial coordination center) and all participating trial sites. All study procedures were performed in accordance with relevant guidelines and written informed consent was obtained from all participating women. The MOBYDIck trial was registered to the ClinicalTrials.gov registry on 25/02/2015 with identifier NCT02371460.

### Study design, population and settings

The MOBYDIck trial was a randomized, double-blind, placebo-controlled clinical trial that took place in 16 neonatal intensive care units in Canada from June 23rd, 2015 until April 3rd, 2018. Mothers who delivered prematurely between 23 0/7 and 28 6/7 weeks of gestation were eligible to participate in the MOBYDIck trial if they intended to breastfeed. Mothers were excluded if they took more than 250 mg/day of DHA supplement during the 3 months prior to delivery. Recruitment of new participants was prematurely terminated by the Data Safety and Monitoring Board on April 4, 2018 due to concern of harm for future participants based on interim data of the risk for BPD favoring the placebo group, findings which were also reported in the N3RO trial^14^.

### Intervention

Eligible and consenting mothers were randomized within 72 h of delivery, to receive 4 capsules per day containing either DHA rich-algae oil providing 1.2 g/day of DHA (S-DHA group) or a placebo containing a mix of corn and soy oils (placebo group) until their infant reached 36 weeks of postmenstrual age. The LC-PUFA-rich oil in the S-DHA capsules was derived from the algae *Schizochytrium* sp. Maternal compliance to the intervention was assessed by counting capsules returned by mothers on postnatal day 14.

### Capsules FA composition

An independent verification of the FA contained in the algae oil and placebo capsules is presented in Table 3. Major FA in algae oil capsules were DHA (45%), n-6 DPA (19%) and 16:0 (17%), while in placebo capsules major FA were LA (52%), *cis-*9 18:1 (26%) and 16:0 (11%).

### Milk sample collection

Breast milk samples were collected on postnatal day 14 in both groups. The date and time of sampling were noted for each sample. Mothers extracted milk from one breast, the milk was then mixed in a bottle and 1 ml was transferred, using a sterile transfer pipette, into identified bar-coded tubes. Samples were put on ice for less than one hour before being frozen at − 80 °C. For sites where − 80 °C freezers were not available, the retention period to − 20 °C was less than 24 h. Throughout the study, samples from each participating site were shipped to the trial coordination center in two batches. For the transport, the box containing the milk samples was placed in a transparent plastic bag containing a sheet of absorbent paper. The sample box was then placed in a cooler, containing dry ice at the bottom, on the sides and at the top to ensure optimal storage. Upon arrival at the site, the integrity of each sample was verified and they were then stored at − 80 °C.

### Breast milk fatty acid analysis

Breast milk samples were analyzed individually for FA composition. With a 0.1 ml sample of milk, lipids were extracted with a C17:0 triacylglycerol as an internal standard (Sigma Aldrich, Oakville, ON, Canada) in 11 ml of chloroform/methanol/salin mixture (2:1:0.85 vol/vol/vol) according to a modified Folch method^46,47^. Total lipids were then methylated using methanol/benzene (4:1 vol/vol) and acetyl chloride^48^. The FA profiles were obtained by capillary gas chromatography using a temperature gradient on an HP 5890 gas chromatograph (Hewlett Packard, Toronto, Canada) equipped with an HP-88 capillary column (100 m × 0.25 mm ID × 0.20 mm film thickness; Agilent Technologies, Santa Clara, CA) coupled with a flame ionization detector as described previously^49^. Helium was used as the carrier gas (split ratio of 1:50). FA were identified according to their retention time using the following standard mixture of FA (FAME 37 mix, Supelco Inc., Bellefonte, PA): C17:0, C22:5 n-6 (Larodan AB, Malmö, Sweden), C22:5 n-3 (Supelco Inc., Bellefonte, PA), C16:0 DMA, C18:0 DMA, C18:1 DMA (Avanti Polar Lipids, Alabaster, AL) and a mixture of 31 FA GLC-411 (NuChek Prep Inc., Elysian, MN). Finally, a mixture of trans-FA containing cis/trans C18:2 n-6 (Supelco Inc., Bellefonte, PA), cis/trans C18:3 n-3 (Supelco Inc., Bellefonte, PA), trans-9 14:1, trans-9 16:1 and isoforms of C18:1 (cis-6, cis-11, cis-12, cis-13, trans-6 and trans-11) (Supelco Inc., Bellefonte, PA) was also used as standard. Results were expressed as % of total FA and the inter and intra-assay error varied between 3.01 and 4.63 in function of the specific FA measured.

### Statistical analysis

The breast milk FA were described as mean ± SD. As the data did not follow a normal distribution, the non-parametric Wilcoxon rank-sum test was done to compare milk FA composition by groups. Generalized linear regression models were fitted to test the influence of maternal DHA capsule compliance, or diet DHA intake on the breast milk DHA level. The results of modelling were presented by regression coefficients (β) and coefficients of determination (r^2^). Statistical analyses were performed using SAS Statistical Software v.9.4 (SAS Institute, Cary, NC, USA) with a two-sided significance level set at P = 0.05.

## Supplementary Information

Supplementary Information.
