# Supplementary material for: Docosahexaenoic acid-rich algae oil supplementation on breast milk fatty acid profile of mothers who delivered prematurely: a randomized clinical trial
Source: Sci Rep. 2021 Nov 2;11:21492. doi: 10.1038/s41598-021-01017-8 (PMC8564506; doi:10.1038/s41598-021-01017-8)
Supplement: Supplementary file 1 — Supplementary Information. [file 41598_2021_1017_MOESM1_ESM.pdf]

Supplementary online content

Docosahexaenoic acid-rich algae oil supplementation on breast milk fatty acid profile of mothers who delivered prematurely: A Randomized Clinical Trial

Hélène Fougère, Jean-François Bilodeau, Pascal M. Lavoie, Ibrahim Mohamed, Iwona Rudkowska, Etienne Pronovost, David Simonyan, Line Berthiaume, Mireille Guillot, Bruno Piedboeuf, Pierre Julien, Isabelle Marc

Supplementary Table S1. Studies, from the literature, reporting breast milk fatty acids composition in response to a supplementation in DHA (alone or in association with other long-chain polyunsaturated fatty acids).

Supplementary Table S1. Studies, from the literature, reporting breast milk fatty acids composition in response to a supplementation in DHA (alone or in association with other long-chain polyunsaturated fatty acids).

Reference	Country	Mothers of preterm	Sample (n)	Nature of oral lipid supplement/amount per day	Placebo	Beginning of treatment	Duration of treatment	Period in which the milk was evaluated	Fatty acids in milk samples of treated	Fatty acids in milk samples of placebo	Response (%)[a]
Valentine et al. (2019) [11]	USA	Yes	10 lactating mothers	Algae (1000mg DHA)	NS (before start supplementation)	The day of birth	4 weeks	Week 4 after delivery	DHA: 230mg/L	DHA: 90mg/L	DHA: +156
Smithers et al. (2010) [21]	Australia	Yes	98 pregnant women	Tuna oil (900mg DHA + 195mg EPA + 54mg AA)	Soy oil	Before delivery	Until expected date of delivery	During the intervention period at 2 weeks intervals	DHA: 1%<br>EPA: 0.1%<br>DPA: 0.2%<br>AA: 0.5%	DHA: 0.3%<br>EPA: 0.08%<br>DPA: 0.2%<br>AA: 0.5%	DHA: +233<br>EPA: +25<br>DPA: =<br>AA: =
Boris et al. (2004) [23]	Denmark	No	21 pregnant women	Fish oil (900mg DHA + 1300mg EPA)	NS	Week 30 of gestation	Until day 30 after delivery	Day 30 after delivery	DHA: 1.4%<br>EPA: 0.7%<br>DPA: 0.6%	DHA: 0.5%<br>EPA: 0.1%<br>DPA: 0.2%	DHA: +180<br>EPA: +600<br>DPA: +200
Jensen et al. (2000) [27]	USA	No	11 lactating women	Eggs (170mg DHA)	NS	Week 2 after delivery	6 weeks	Week 8 after delivery	DHA: 0.29%<br>EPA: 0.06%<br>n-6 DPA: 0.05%<br>AA: 0.46%	DHA: 0.22%<br>EPA: 0.07%<br>n-6 DPA: 0.09%<br>AA: 0.61%	DHA: +32<br>EPA: (-14)<br>n-6 DPA: -44<br>AA : (-25)
Jensen et al. (2000) [27]	USA	No	11 lactating women	Fish oil (260mg DHA)	NS	Week 2 after delivery	6 weeks	Week 8 after delivery	DHA: 0.39%<br>EPA: 0.09%<br>n-6 DPA: 0.12%<br>AA: 0.55%	DHA: 0.27%<br>EPA: 0.08%<br>n-6 DPA: 0.08%<br>AA: 0.60%	DHA: +44<br>EPA : (+13)<br>n-6 DPA: +50<br>AA: (-8)
Much et al. (2013) [28]	Germany	No	152 pregnant women	1020mg DHA + 180mg EPA	NS	Week 15 of gestation	Until month 4 after delivery	Week 6 after delivery<br><br><br><br>Week 16 after delivery	DHA: 1.34%<br>EPA: 0.18%<br>DPA: 0.23%<br>AA: 0.43%<br>DHA: 1.12%<br>EPA: 0.15%<br>DPA: 0.21%<br>AA: 0.40%	DHA: 0.28%<br>EPA: 0.08%<br>DPA: 0.17%<br>AA: 0.43%<br>DHA: 0.24%<br>EPA: 0.07%<br>DPA: 0.16%<br>AA: 0.40%	DHA: +379<br>EPA: +125<br>DPA: +35<br>AA: =<br>DHA: +367<br>EPA: +114%<br>DPA: +31<br>AA: =
Helland et al. (1998) [24]	Norway	No	5 lactating women	2.5mL cod liver oil (255mg DHA)	NS	Week 3 to-8 after delivery	14 days	Day 14	DHA: 0.49%<br>EPA: 0.28%<br>DPA: 0.14%<br>AA: 0.31%	DHA: 0.36%<br>EPA: 0.14%<br>DPA: 0.11%<br>AA: 0.34%	DHA: +36<br>EPA: (+100)<br>DPA: (+27)<br>AA: (-9)
Helland et al. (1998) [24]	Norway	No	5 lactating women	5mL cod liver oil (511mg DHA)	NS	Week 3 to 8 after delivery	14 days	Day 14	DHA: 0.59%<br>EPA: 0.28%<br>DPA: 0.18%<br>AA: 0.32%	DHA: 0.33%<br>EPA: 0.11%<br>DPA: 0.10%<br>AA: 0.28%	DHA: +79<br>EPA: +155<br>DPA: (+800<br>AA: (+14)
Helland et al. (1998) [24]	Norway	No	6 lactating women	10mL cod liver oil (1021mg DHA)	NS	Week 3 to 8 after delivery	14 days	Day 14	DHA: 1.11%<br>EPA: 0.43%<br>DPA: 0.22%<br>AA: 0.36%	DHA: 0.37%<br>EPA: 0.16%<br>DPA: 0.12%<br>AA: 0.32%	DHA: +200<br>EPA: +169<br>DPA: +83<br>AA: (+13)
Cimatti et al. (2018) [37]	Italy	No	16 lactating mothers	Krill oil (250mg DHA + 70mg EPA)	NS	Week 4 to 6 after delivery	30 days	Day 30	DHA: 0.42%<br>EPA: 0.11%<br>AA: 0.43%	DHA: 0.28%<br>EPA: 0.10%<br>AA: 0.60%	DHA: +50<br>EPA: +10<br>AA: -28[b]
Bergmann et al. (2008) [50]	Germany	No	53 lactating mothers	Low EPA fish oil (200mg DHA)	Vitamin and mineral supplement	Week 2 after delivery	45 days	Month 3 after delivery	DHA: 0.50%<br>EPA: 0.13%<br>AA: 0.48%	DHA: 0.25%<br>EPA: 0.09%<br>AA: 0.47%	DHA: +100<br>EPA: +44<br>AA: (+2)
Dunstan et al. (2007) [51]	Australia	No	73 pregnant women	Fish oil (1000mg)	Olive oil	Week 20 of gestation	Until delivery	Day 3 after delivery	DHA: 1.15%<br>EPA: 0.16%<br>DPA: 0.56%<br>AA: 0.55%	DHA: 0.5%<br>EPA: 0.06%<br>DPA: 0.28%<br>AA: 0.61%	DHA: +130<br>EPA: +167<br>DPA: +100<br>AA: (-10)

								Week 6 after delivery<br><br><br><br>Month 6 after delivery	DHA: 0.42%<br>EPA: 0.09%<br>DPA: 0.20%<br>AA: 0.35%<br>DHA: 0.38%<br>EPA: 0.10%<br>DPA: 0.21%<br>AA: 0.36%	DHA: 0.25%<br>EPA: 0.07%<br>DPA: 0.17%<br>AA: 0.38%<br>DHA: 0.34%<br>EPA: 0.10%<br>DPA: 0.22%<br>AA: 0.37%	DHA: +68<br>EPA : +29<br>DPA: (+18)<br>AA: (-8)<br>DHA: (+12)<br>EPA: =<br>DPA: (+5)<br>AA: (-3)
Fidler et al. (2000) [52]	Germany	No	10 lactating women	200mg DHA	NS	Week 4 after delivery	2 weeks	Day 14	DHA: 0.37%<br>EPA: 0.05%<br>DPA: 0.15%<br>AA: 0.41%	DHA: 0.21%<br>EPA: 0.05%<br>DPA: 0.15%<br>AA: 0.43%	DHA: +76<br>EPA: =<br>DPA: =<br>AA: (-5)
Hurtado et al. (2015) [53]	Spain	No	76 pregnant women	Fish oil drink (320mg DHA + 72mg EPA)	NS	Week 28 of gestation	Until the end of lactation	Colostrum<br><br>1 month's milk<br><br>2 month's milk<br><br>4 month's milk	DHA: 1.03%<br>EPA: 0.22%<br>DHA: 0.67%<br>EPA: 0.18%<br>DHA: 0.80%<br>EPA: 0.18%<br>DHA: 0.65%<br>EPA: 0.17%	DHA: 0.67%<br>EPA: 0.12%<br>DHA: 0.41%<br>EPA: 0.12%<br>DHA: 0.37%<br>EPA: 0.11%<br>DHA: 0.32%<br>EPA: 0.08%	DHA: +54<br>EPA: +83<br>DHA: +63<br>EPA: +50<br>DHA: +116<br>EPA: +64<br>DHA: +103<br>EPA: +113
Imhoff-Kunsch et al. (2011) [54]	Mexico	No	174 pregnant women	400mg DHA	NS	Week 18 to 22 of gestation	Until delivery	Month 1 after delivery	DHA: 0.20%<br>EPA: 0.14%<br>DPA: 0.10%<br>AA: 0.41%	DHA: 0.17%<br>EPA: 0.16%<br>DPA: 0.12%<br>AA: 0.43%	DHA: +18<br>EPA: (-13)<br>DPA: (-17)<br>AA: (-5)
Sherry et al. (2015) [55]	USA	No	56 lactating mothers	200mg DHA	NS	Week 4 to 6 after delivery	6 weeks	Week 6	DHA: 88.3mg/L<br>EPA: 19mg/L<br>DPA: 34.3mg/L<br>AA: 115.8mg/L	DHA: 58.7mg/L<br>EPA: 16.1mg/L<br>DPA: 39.9mg/L<br>AA: 148.3mg/L	DHA: +50<br>EPA: (+18)<br>DPA: (-14)<br>AA: (-22)
Sherry et al. (2015) [55]	USA	No	53 lactating mothers	400mg DHA	NS	Week 4 to 6 after delivery	6 weeks	Week 6	DHA: 130.8mg/L<br>EPA: 20.6mg/L<br>DPA: 35.8mg/L<br>AA: 121.2mg/L	DHA: 58.7mg/L<br>EPA: 16.1mg/L<br>DPA: 39.9mg/L<br>AA: 148.3mg/L	DHA: +123<br>EPA: (+28)<br>DPA: (-10)<br>AA: (-18)
Smit et al. (2000) [56]	Holland	No	16 lactating women	400mg DHA + 300mg AA + 110mg EPA	NS	During breastfeeding (Month 3 to 10)	1 week	Day 1<br><br><br><br>Day 7	DHA: 0.25%<br>EPA: 0.07%<br>n-6 DPA: 0.05%<br>AA: 0.61%<br>DHA: 0.21%<br>EPA: 0.06%<br>n-6 DPA: 0.05%<br>AA: 0.69%	DHA: 0.20%<br>EPA: 0.05%<br>n-6 DPA: 0.05%<br>AA: 0.55%<br>DHA: 0.20%<br>EPA: 0.05%<br>n-6 DPA: 0.05%<br>AA: 0.55%	DHA: (+25)<br>EPA: +40<br>n-6 DPA: =<br>AA: (+11)<br>DHA: (+5)<br>EPA: (+20)<br>n-6 DPA: =<br>AA: (+26)
Urwin et al. (2012) [57]	UK	No	52 pregnant women	Salmon (480mg EPA + DHA)	Habitual diet	Week 20 of gestation	Until birth	Day 1 after delivery<br><br><br><br>Day 5 after delivery<br><br><br><br>Day 14 after delivery	DHA: 0.66%<br>EPA: 0.09%<br>DPA: 0.45%<br>AA: 0.88%<br>DHA: 0.65%<br>EPA: 0.11%<br>DPA: 0.30%<br>AA: 0.60%<br>DHA: 0.50%<br>EPA: 0.10%<br>DPA: 0.22%<br>AA: 0.52%	DHA: 0.49%<br>EPA: 0.06%<br>DPA: 0.33%<br>AA: 0.98%<br>DHA: 0.35%<br>EPA: 0.06%<br>DPA: 0.23%<br>AA: 0.62%<br>DHA: 0.31%<br>EPA: 0.08%<br>DPA: 0.20%<br>AA: 0.56%	DHA: (+35)<br>EPA: (+50)<br>DPA: +36<br>AA: (-10)<br>DHA: +86<br>EPA: +83<br>DPA: +30<br>AA: (-3)<br>DHA: +61<br>EPA: (+25)<br>DPA: (+10)<br>AA: (-7)

								Day 28 after delivery	DHA: 0.43%<br>EPA: 0.09%<br>DPA: 0.27%<br>AA: 0.49%	DHA: 0.32%<br>EPA: 0.07%<br>DPA: 0.23%<br>AA: 0.53%	DHA: +34<br>EPA: (+29)<br>DPA: +17%<br>AA: (-8)
Van Goor et al. (2009) [58]	Holland	No	48 pregnant women	DHA (220mg)	Soy bean oil	Week 14 to 20 of gestation	Until week 12 after delivery	Week 2 after delivery<br><br>Week 12 after delivery	DHA: 0.60%<br>AA: 0.57%<br>DHA: 0.39%<br>AA: 0.39%	DHA: 0.40%<br>AA: 0.58%<br>DHA: 0.25%<br>AA: 0.39%	DHA: +50<br>AA: (-2)<br>DHA: +56<br>AA: =
Van Goor et al. (2009) [58]	Holland	No	37 pregnant women	DHA + AA (220mg + 220mg)	Soy bean oil	Week 14 to 20 of gestation	Until week 12 after delivery	Week 2 after delivery<br><br>Week 12 after delivery	DHA: 0.51%<br>AA: 0.66%<br>DHA: 0.38%<br>AA: 0.49%	DHA: 0.40%<br>AA: 0.58%<br>DHA: 0.25%<br>AA: 0.39%	DHA: +28<br>AA: +14<br>DHA: +52<br>AA: (+26)
Weseler et al. (2008) [59]	Holland	No	16 lactating women	320mg DHA + 80mg EPA	NS	Week 3 after delivery	2 weeks<br><br>8 weeks	Week 5 after delivery<br><br>Week 11 after delivery	DHA: 0.55%<br>AA: 0.43%<br>DHA: 0.53%<br>AA: 0.40%	DHA: 0.25%<br>AA: 0.45%<br>DHA: 0.24%<br>AA: 0.41%	DHA: +120<br>AA: (-4)<br>DHA: +121<br>AA: (-2)

[a] Responses reported when treatment effects were significant (P<0.05) and calculated as [((Treatment – Control)/Control) x 100]. Values in parentheses are not significant (P > 0.05).

[b] Values where P-values were not specified.

AA: arachidonic acid, DHA: docosahexaenoic acid, DPA: docosapentaenoic acid, EPA: eicosapentaenoic acid, NS: Not supplemented.
